# The Antimalarial Drug Artesunate Mediates Selective Cytotoxicity by Upregulating HO-1 in Melanoma Cells

**DOI:** 10.3390/biomedicines11092393

**Published:** 2023-08-27

**Authors:** Finn Jochims, Rebecca Strohm, Claudia von Montfort, Chantal-Kristin Wenzel, Niklas Klahm, Arun Kumar Kondadi, Wilhelm Stahl, Andreas S. Reichert, Peter Brenneisen

**Affiliations:** Institute of Biochemistry and Molecular Biology I, Medical Faculty, Heinrich Heine University Düsseldorf, 40225 Düsseldorf, Germany; rebecca.strohm@hhu.de (R.S.); chantal-kristin.wenzel@hhu.de (C.-K.W.); niklas.klahm@hhu.de (N.K.); arun.kondadi@hhu.de (A.K.K.); wilhelm.stahl@hhu.de (W.S.); reichert@hhu.de (A.S.R.)

**Keywords:** melanoma, cancer therapy, drug repurposing, oxidative stress, natural compound, artesunate, fibroblasts, heme oxygenase 1, iron

## Abstract

Despite great efforts to develop new therapeutic strategies to combat melanoma, the prognosis remains rather poor. Artesunate (ART) is an antimalarial drug displaying anti-cancer effects in vitro and in vivo. In this in vitro study, we investigated the selectivity of ART on melanoma cells. Furthermore, we aimed to further elucidate the mechanism of the drug with a focus on the role of iron, the induction of oxidative stress and the implication of the enzyme heme oxygenase 1 (HO-1). ART treatment decreased the cell viability of A375 melanoma cells while it did not affect the viability of normal human dermal fibroblasts, used as a model for normal (healthy) cells. ART’s toxicity was shown to be dependent on intracellular iron and the drug induced high levels of oxidative stress as well as upregulation of HO-1. Melanoma cells deficient in HO-1 or treated with a HO-1 inhibitor were less sensitive towards ART. Taken together, our study demonstrates that ART induces oxidative stress resulting in the upregulation of HO-1 in melanoma cells, which subsequently triggers the effect of ART’s own toxicity. This new finding that HO-1 is involved in ART-mediated toxicity may open up new perspectives in cancer therapy.

## 1. Introduction

More than half a century after the first chemotherapy, cancer is still the second leading cause of deaths worldwide [1]. Malignant melanoma represents the most aggressive type of skin cancer with more than 300,000 diagnosed cases worldwide and an incidence which is predicted to be further rising [2]. Despite the ever-increasing need for new and more selective treatment options, the rate of new approved drugs remains very low [3]. This is partly due to the time-consuming and cost-intensive nature of the development and approval process, finally resulting in high failure rates [4]. In addition to an often applied monotargeted therapy, “drug repurposing” is a recently rediscovered and rather systemically oriented therapeutic approach, involving the use of already approved drugs for new indications. This concept can increase the success rate because the toxicological and pharmacological characteristics of the respective drugs have already been well described [5]. One such drug to be repurposed and used for tumor therapy might be artesunate (ART). Decades of clinical experience have shown almost no adverse effects inherent in this antimalarial drug [6,7]. Previous studies demonstrated anti-cancer effects of ART in vitro and in vivo. For instance, ART was shown to induce apoptosis in breast cancer cells [8], ferroptosis in pancreatic ductal adenocarcinoma cells [9] and necroptosis in schwannoma cells [10]. Furthermore, the drug was shown to interfere with angiogenesis and invasion in human cancer cells [11,12]. Moreover, first clinical phase I trials in patients with metastatic breast cancer were conducted and no major safety concerns were observed when administering long-term oral ART [13,14]. However, the exact underlying molecular mechanism and the proteins involved are still not fully explained.

Reactive oxygen species (ROS) are highly reactive molecules generated as by-products in normal cellular activity, with mitochondria being the main ROS-producing organelle [15]. While physiological concentrations of ROS are essential for redox signaling (termed “oxidative eustress”), supraphysiological concentrations are capable of damaging intracellular macromolecules, finally resulting in cell death (termed “oxidative distress”) [16]. In almost all cancer cells, an increase in ROS was detected [17] which is compensated by increased levels of antioxidative proteins [18]. Impairing this delicate balance represents an attractive target for selective anti-cancer therapy as normal (healthy) cells are less vulnerable to the induction of oxidative stress [18,19,20]. In this context, a pro-oxidative approach has already been proven successful in several in vitro studies [21,22,23]. The anti-cancer effects of ART are also associated with increased ROS as measured using the ROS indicators H_2_DCFDA or dihydroethidium [24,25].

Alongside higher ROS levels, cancer cells display an abnormal iron homeostasis and compromise increased levels of intracellular iron [26,27,28]. Involved in numerous redox reactions the divalent metal is critical for survival functions, whereas its redox-modulatory abilities also facilitate the production of highly toxic radicals (e.g., through Fenton reaction). On the basis of this dual role of iron, a system evolved in numerous living organisms which tightly controls iron homeostasis [29]. The majority of intracellular iron (~95%) is protein-bound and only a small proportion is bound to low-molecular-weight substances or unbound [30]. This proportion of chelatable, redox-active and mostly reduced iron (Fe^2+^) is described as “labile iron pool” (LIP) and is, contrarily to the protein-bound iron, highly active [31]. A major source of Fe^2+^ within the LIP is heme oxygenase 1 (HO-1) [32]. The human HO-1 is an inducible enzyme which expression is regulated by various transcription factors such as Nrf2, MAPK, AP-1 and NF-ĸb [33]. HO-1 catalyzes the first and rate-limiting step of heme degradation, releasing biliverdin, carbon monoxide (CO) and Fe^2+^ [34]. While the enzyme is usually associated with a cytoprotective role, augmentation of the LIP also indicates a potential deleterious effect [35].

In this study, the selective effect of ART on melanoma cells in comparison to normal (healthy) cells was investigated, with a special focus on the induction of oxidative stress and the role of Fe^2+^ releasing HO-1. Apart from demonstrating selective cytotoxicity and induction of oxidative stress, it was shown for the first time that HO-1 may play a role in the ART mediated toxicity on melanoma cells.

## 2. Materials and Methods

### 2.1. Materials

The chemicals used and Dulbecco’s modified Eagle’s medium (DMEM) were purchased from Sigma-Aldrich (Taufkirchen, Germany) or Merck (Darmstadt, Germany), unless otherwise stated. Artesunate (CAS 88495-63-0) was obtained from Selleck Chemicals GmbH (Planegg, Munich, Germany). Fetal bovine serum (FBS) was acquired from Pan-Biotech (Aidenbach, Germany). Penicillin/Streptomycin was purchased from Biochrom (Berlin, Germany) and Glutamax^TM^ from Gibco (Darmstadt, Germany). Hemin (CAS 16009-13-5), Deferoxamine mesylate salt (CAS 138-14-7) and DCFH-DA (CAS 4091-99-0) were obtained from Sigma-Aldrich (St. Louis, MO, USA). Zinc protoporphyrin (CAS 15442-64-5) was acquired from Enzo Life Science (Lörrach, Germany). All reagents for flow cytometric analysis were obtained from Sysmex-Partec (Kobe, Japan). DC™ protein assay kit was purchased from Bio-Rad (Feldkirchen, Germany). Clarity™ Western ECL Substrate was obtained from Bio-Rad Laboratories, Inc., (Hercules, CA, USA).

### 2.2. Cell Culture

Human melanoma cell line A375 (CRL-1619) was purchased from the American Type Culture Collection (ATCC, Manassas, VA, USA). Normal human dermal fibroblasts (NHDF; C-12300) were purchased from PromoCell (Heidelberg, Germany). The cultivation and treatment of the cells were carried out under sterile conditions. The cell lines used, A375 and NHDF, were cultured in Dulbecco’s modified Eagle’s medium (DMEM, low glucose), including 10% FBS, GlutaMAX™ (2 mM), penicillin (100 U/mL) and streptomycin (100 µg/mL). For treatment, the cells were cultured until subconfluence (70–80%), washed with PBS and subsequently treated with the respective substances dissolved in DMEM with 4500 mg/L glucose (high glucose, HG), supplemented with GlutaMAX™ (2 mM), penicillin (100 U/mL) and streptomycin (100 µg/mL).

### 2.3. Viability—Sulforhodamine B Assay (SRB-Assay)

This assay is based on the pH-dependent binding of SRB to positively charged amino acids of cellular proteins [36]. The cells were incubated in 24-well plates and treated with different concentrations of ART for the indicated time. After treatment, the cells were washed once with PBS and fixed by adding 500 µL of TCA (10% *w*/*v*) for 1 h at 4 °C and washing 5× with dH_2_O. Subsequently, the cells were incubated with 300 µL of SRB (0.4% *w*/*v* in 1% acetic acid) for 15 min at RT, washed 5x with acetic acid (1% *v*/*v*) and dried overnight. For quantification of the protein content per well which is proportional to the number of surviving cells per well, 400 µL of TRIS (10 mmol/L) was added to each well. Finally, the absorption was measured at 492 nm against a background of 620 nm with a microplate reader (Tecan M200 pro, Männedorf, Switzerland). The mean optical density (OD) of the H_2_O_2_ positive treatment was subtracted from all other OD values. Cell viability of mock-treated cells was set at 100%.

### 2.4. ROS Measurement via FACS

To assess changes in the redox state of the cell, the widely used fluorescent probe 2′,7′-dichlorodihydrofluorescein (DCFH) was administered in its diacetate form (DCFH-DA). The small molecule itself is non-fluorescent but after diffusion into the cell and cleavage of the ester bond using cellular esterases, it can be oxidized via ROS to a fluorescent product (DCF). This reaction is not specific to a particular kind of ROS. However, it can be used to determine general changes in the redox state [37]. Cells were cultivated in Ø6 cm dishes for 24 h, washed once with PBS and treated for 30 min with DCFH-DA (10 µM). After washing again 1x with PBS the cells were treated with given concentrations of ART for 1 h, drained with 500 mL Trypsin/EDTA and collected in FCS-containing DMEM (LG), followed by washing 2× with PBS. The fluorescence intensity of oxidized DCF was measured by using the flow cytometer Cube6 (Sysmex-Partec, Kobe, Japan). The selected excitation and emission wavelengths were 488 nm and 536 ± 20 nm, respectively (fluorescent channel 1, FL1). The resulting data were analyzed with the FlowJo v.10.7.2 software (BD Biosciences, Franklin Lakes, NJ, USA).

### 2.5. SDS-PAGE and Western Blot

For analysis of protein expression in mock-treated and ART-treated cells, sodium dodecyl sulfate polyacrylamide gel electrophoresis (SDS-PAGE) and subsequent western blotting were performed [38]. Treated cells were lysed in 1% SDS with 1:1000 protease inhibitor cocktail (Sigma, Taufkirchen, Germany). After sonification (Branson Sonifier 250, Branson Ultrasonics, Brookfield, WI, USA) of the lysates, the protein concentration was determined by using the DC^TM^ Protein Assay Kit. Before SDS-PAGE, 20 µg of protein lysate was incubated with 4× sample puffer (40% glycerol, 20% ß-mercaptoethanol, 12% SDS, 0.4% bromphenol blue) and heated at 95 °C for 10 min. After 12% (*w*/*v*) SDS-PAGE, the proteins were electroblotted onto a polyvinylidene difluoride (PVDF) membrane (GE Healthcare, Solingen, Germany). Blocking of unspecific binding sites was carried out for 1 h in milk powder (5% (*w*/*v*)) and incubation with the primary antibody was performed overnight. Subsequently, the membrane was washed 3× with TBST, incubated for 1 h with the secondary antibody and again washed 3x with TBST. Visualization of the proteins was performed by using the ECL-system (Cell Signaling Technology) and monitored using the Fusion SL Advance gel documentation device (Peqlab, Erlangen, Germany). Quantification of proteins was carried out using the FusionCapt Advance software (Vilber Lourmat, Eberhardzell, Germany).

The following primary antibodies were used in 1:1000 dilution: recombinant anti-heme oxygenase 1 antibody [EP1391Y] (ab52947) rabbit mAb (Abcam, Cambridge, UK), β-tubulin antibody (9F3) rabbit mAb (#2128) (Cell Signaling Technology, Danvers, Beverly, MA, USA). The following secondary antibodies were used in 1:10,000 dilution: goat IgG anti-rabbit IgG (H+L)-HRPO pAb (#111-035-144) (Dianova, Hamburg, Germany).

### 2.6. Generating HO-1 Knock Out Cell Line

For generating a HO-1 knock out cell line, double nickase plasmids from Santa Cruz (sc-400157-NIC) were used. The plasmids encode for Cas9 and two different gRNAs, which bind on both sides of the DNA strand, close together, creating a double-strand break. One plasmid also encodes for GFP, the other for puromycin resistance, resulting in different selection options. At first, 100k A375 cells were seeded in a 3 cm dish and grown overnight to about 50% confluency. On the next day, a transfection with the plasmids was carried out according to the GeneJuice manufacturers’ guide. The cells were grown for three days in the transfection medium and then harvested with cell dissociation buffer. Single cells in a 96-well format were obtained via FACS sorting, using GFP as a marker for transfection. After growing confluent, the obtained colonies were moved into bigger formats over time and a validation of the KO was carried out using Western blot with hemin as an inducer of HO-1. The validated clone was frozen and used for further experiments.

### 2.7. Statistical Analysis

Data were presented as mean ± standard error of the mean (SEM). Mean values were calculated from at least three independent experiments (*n* ≥ 3), unless stated otherwise. Analysis of significance was performed using one-way ANOVA with post hoc test (Dunnett’s test) or Student’s *t*-test (α = 0.05) using the software Graph Pad Prism 9.1.1 (GraphPad Software, San Diego, CA, USA). Levels of significance were defined as * *p* < 0.05, ** *p* < 0.01 and *** *p* < 0.001.

## 3. Results

### 3.1. ART Displays Selective Cytotoxicity on Cancer Cells Depending on Intracellular Iron (Fe^2+^)

**Figure 1:**
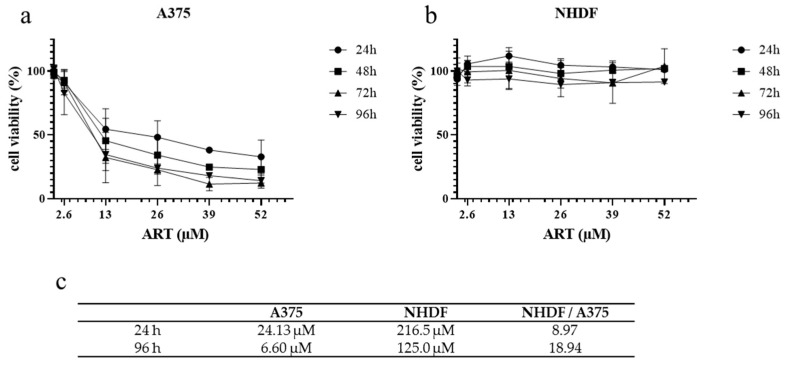
Effect of ART on cell viability in A375 and NHDF cells. To detect ART’s influence on cell viability, A375 (**a**) and NHDF cells (**b**) were treated with concentrations from 2.6 to 52 µM for 24–96 h. Cell viability was measured via SRB assay. DMSO (0.1%)-treated control was set at 100%. Data represent means ± SEM of three independent experiments (*n* = 3). (**c**) IC_50_-values of ART in melanoma cells and fibroblasts were calculated via non-linear curve fit analysis using the Prism software (GraphPad, San Diego, CA, USA). In addition, the therapeutic index (NHDF/A375) was calculated.

The potential of a novel anti-cancer drug is highly dependent on its selectivity on cancer cells without affecting non-malignant (healthy) cells. Therefore, the cytotoxicity of ART was tested on A375 melanoma cells in comparison to NHDF (normal human dermal fibroblasts), used as a model for normal (healthy) cells. According to a study performed in breast cancer cells [8], ART was used in concentrations from 2.6 to 52 µM (equivalent to 1 µg/mL–20 µg/mL). ART treatment of A375 melanoma cells decreased cell viability in a time- and dose-dependent manner (Figure 1a). Contrary to the toxic effect on A375 cells, ART concentrations up to 52 µM did not affect cell viability of normal NHDF (Figure 1b). Therefore, no time or dose dependency was observed. For calculating IC_50_-values, NHDF were treated with ART concentrations up to 300 µM. The IC_50_-values of ART for 24 h and 96 h treatment were distinctly lower in A375 cells (24.13 µM and 6.6 µM) than in NHDF (216.5 µM and 125 µM; Figure 1c). In an approach analogous to calculating the therapeutic index in vivo, the ratio of IC_50_-values of NHDF and A375 was calculated, showing an in vitro therapeutic index of 8.97 (24 h) or 18.94 (96 h).

**Figure 2:**
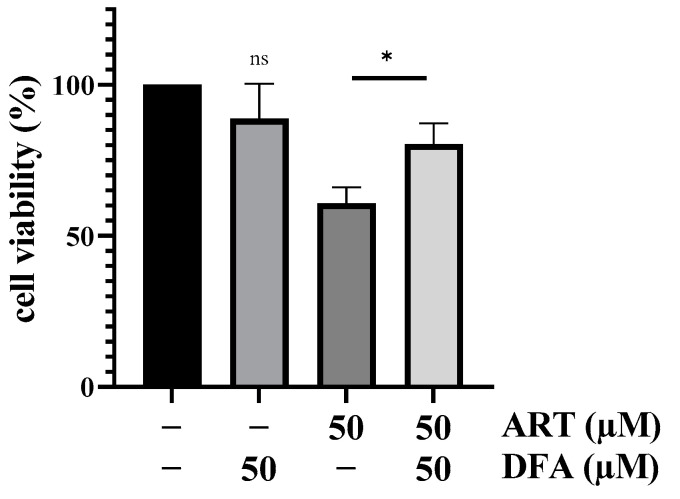
Effect of iron on ART-mediated cytotoxicity on melanoma cells. A375 melanoma cells were either pre-treated with DFA (50 µM) or did not receive pre-treatment and were subsequently incubated with ART (50 µM) for 24 h. Cell viability was measured via SRB assay. DMSO (0.2%)-treated control was set at 100%. Data represent means ± SEM of three independent experiments (*n* = 3). The level of significance was calculated with Student’s *t*-test with ns ≥ 0.05 and * *p* < 0.05.

Subsequently, we investigated ART’s mechanism in melanoma cells in which the drug was particularly toxic. Although the exact mechanism still remains elusive, it is well-accepted from malaria and cancer research that ART is activated in a Fe^2+^-catalyzed reaction resulting in a carbon-centered radical (note that it is still highly controversial if heme-bound Fe^2+^ or free Fe^2+^ or both are mainly responsible for the activation of the drug) [39,40,41]. In order to analyze ART’s dependency on Fe^2+^ in melanoma cells, the iron-chelating agent deferoxamine (DFA) was administered. A375 cells were pre-treated for 4 h with subtoxic concentrations of DFA (50 µM) and afterwards treated for 24 h with 50 µM ART, a concentration known to be highly toxic in A375 cells (Figure 1a). Cells co-treated with ART and DFA displayed significantly higher viability (~ 80%) than cells treated with ART alone (~ 60%; *p* < 0.05; Figure 2). This rescue effect indicated that ART-induced cytotoxicity in human A375 melanoma cells was dependent on the intracellular Fe^2+^-concentration.

### 3.2. ART Selectively Induces Oxidative Stress in Melanoma Cells

**Figure 3:**
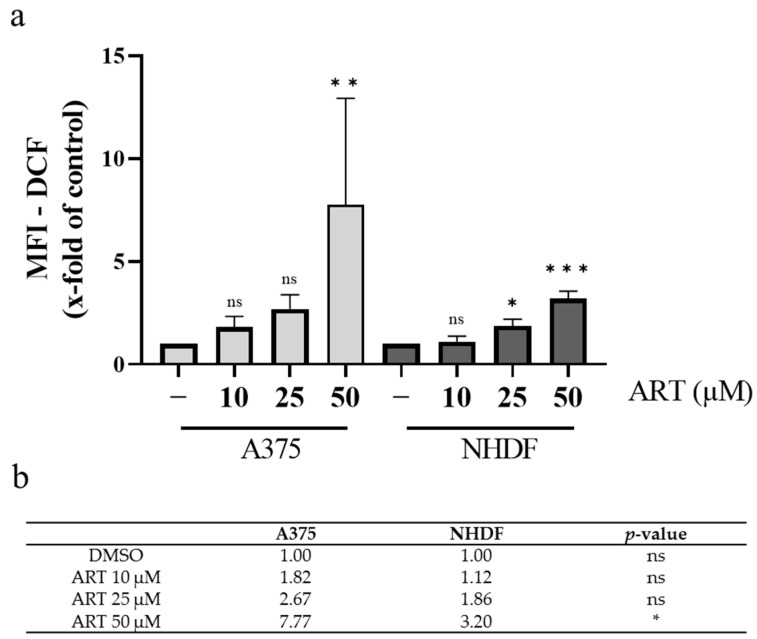
Effect of ART-mediated ROS generation on A375 and NHDF. (**a**) Cells were incubated with DCFH-DA (10 µM) for 30 min and subsequently treated with the given concentrations of ART. The median fluorescence intensity (MFI) of DCF was detected via FACS (Cube 6, Sysmex-Partec, Kobe, Japan) and is stated as x-fold of DMSO (0.1%) control which was set at 1.0. The level of significance was calculated with ANOVA (Dunnett’s test) with ns ≥ 0.05; * *p* < 0.05; ** *p* < 0.01; and *** *p* < 0.001 compared to control (-). (**b**) The MFI-DCF values (x-fold of control) from (**a**) and statistical significance comparing A375 and NHDF are displayed. The level of significance was calculated with two-way ANOVA with ns ≥ 0.05 and * *p* < 0.05.

Several studies link the toxicity of ART to an induction of oxidative distress [8,24,42]. The excessive increase in pro-oxidative molecules, induced by the antimalarial drug was shown to be causative for cell death in numerous cancer cell lines, such as leukemia cells [43] and glioblastoma cells [44]. The impact of ART on the redox state of non-malignant cells, however, is less well-investigated. Therefore, this study aimed to elucidate the selectivity of ART in the context of affecting the intracellular ROS level by comparing A375 and NHDF cells. Both cell lines were incubated with the fluorescent probe DCFH-DA (10 µM) for 30 min and subsequently treated for 1 h with the given concentrations of ART. The fluorescence intensity of the produced DCF was measured via FACS and is stated as x-fold of the respective DMSO control with intracellular statistical analysis (Figure 3a). The same concentrations of ART induced higher levels of ROS in A375 cells than in NHDF. A concentration of 10 µM led to a 1.8-fold increase compared to the control in A375, whereas in NHDF a 1.1-fold increase was observed. Similar results were obtained with 25 µM of ART (2.7 vs. 1.9) and 50 µM of ART (7.8 vs. 3.2). In addition, an intercellular analysis comparing ROS induction between A375 and NHDF cells upon ART treatment detected a significantly higher induction of ROS in A375 than in NHDF for the highest ART concentration (Figure 3b). Therefore, these findings strongly suggested that ART probably induced oxidative distress in malignant and non-malignant cells. However, a tendency towards a higher amount of ROS increase in cancer cells than in normal (healthy) cells was observed.

### 3.3. HO-1 Is Upregulated upon ART Treatment in Melanoma Cells

**Figure 4:**
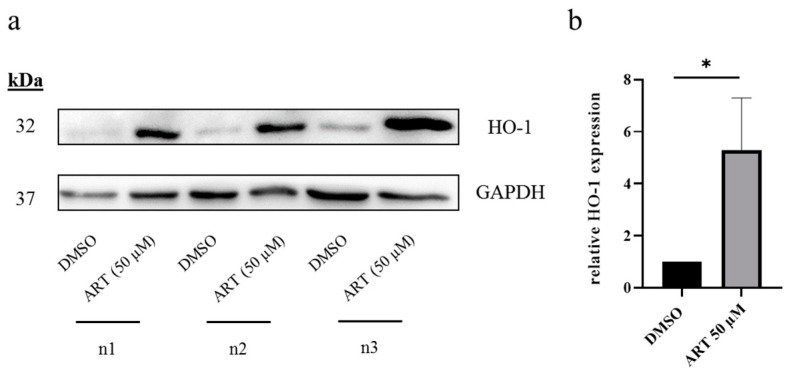
Modulation of HO-1 expression by ART. (**a**) A375 melanoma cells were treated with DMSO (0.1%) or ART (50 µM) for 24 h. Subsequently, the cells were lysed and subjected to Western blot analysis. Detection of HO-1 as well as loading control GAPDH was performed with the Fusion SL Advance gel documentation device (Peqlab, Erlangen, Germany). All three independent experiments (*n* = 3) are shown. (**b**) Protein quantification was performed with Fusion SL Advance gel documentation device. The ratio between the HO-1 signal and the GAPDH signal was calculated and compared to DMSO-treated cells. DMSO-treated control was set at 1.0. Data represent means ± SEM of three independent experiments (*n* = 3). The level of significance was calculated with Student’s *t*-test with * *p* < 0.05.

Following the detection of an ART-mediated increase in ROS, we aimed to confirm this increase by analyzing the expression of antioxidative proteins such as heme oxygenase 1 (HO-1). HO-1 upregulation is regarded as a typical marker for an increase in intracellular ROS levels [45]. The expression of the enzyme was detected via Western blot. For that, A375 cells were treated for 24 h with ART (50 µM) and the expression of HO-1 is shown in comparison to DMSO-treated (mock-treated) cells (Figure 4a). Protein quantification and subsequent statistical analysis of three independently performed experiments (*n* = 3) detected a significant increase in HO-1 expression upon ART treatment (Figure 4b). These data confirmed the previously demonstrated effects of ART on the induction of oxidative stress in A375 cells (see Figure 3).

### 3.4. HO-1 Further Increases ART Mediated Cytotoxicity in Melanoma Cells

**Figure 5:**
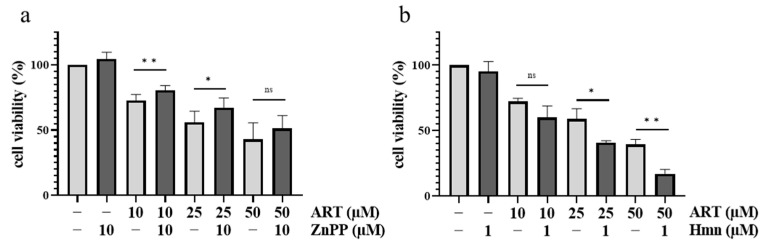
Pharmacological modulation of HO-1 and its effects on ART’s toxicity in melanoma cells. A375 cells were co-treated with ART and ZnPP (**a**) or Hmn (**b**) for 24 h. Cell viability was measured via SRB assay. DMSO (0.2%)-treated control was set at 100%. Data represent means ± SEM of at least three independent experiments (*n* ≥ 3). The level of significance was calculated with Student’s *t*-test with ns ≥ 0.05; * *p* < 0.05 and ** *p* < 0.01.

A closer look on the reaction catalyzed by HO-1 reveals that the enzyme is responsible for degrading heme into biliverdin, carbon monoxide (CO) and Fe^2+^ [34]. Especially the release of Fe^2+^ is very intriguing, as previously shown DFA data (Figure 2) indicated that the divalent metal ion is important for the activation of ART. This gave rise to the questions whether the Fe^2+^-releasing effect of HO-1 may be responsible for an increase in ART toxicity and whether ART could increase its own toxicity by upregulating HO-1. To test this hypothesis, the HO-1 in melanoma cells was modulated with the HO-1-inducing agent hemin (Hmn) and the HO-1-inhibiting agent Zinc protoporphyrin (ZnPP) in the presence of ART [46,47]. A375 cells were co-treated with ZnPP (10 µM) or Hmn (1 µM), and indicated concentrations of ART. In cells co-treated with ZnPP, ART lowered viability to a lesser extent than in cells solely treated with the same concentrations of the drug (Figure 5a). The opposite effect was observed in cells co-treated with Hmn, where ART was more toxic than in cells without HO-1 modulation (Figure 5b). In summary, the modulation of HO-1 by ZnPP and Hmn in combination with ART treatment indicated that the toxicity of the drug in melanoma cells is dependent on the HO-1.

**Figure 6:**
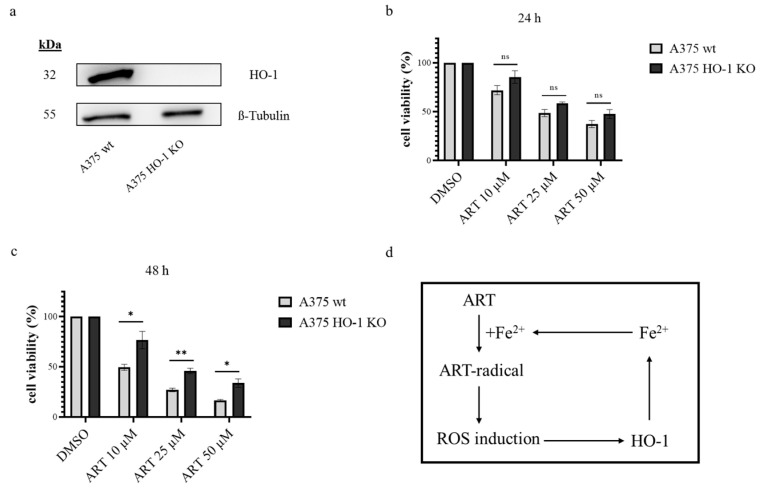
Comparison of ART-mediated cytotoxicity in melanoma cells deficient in HO-1 and wt melanoma cells. (**a**) Western blot comparing the expression of HO-1 and loading control ß-Tubulin in A375 wt and A375 HO-1 KO cells. Both cell lines were treated with Hmn (1 µM) for 24 h to induce HO-1 expression. (**b**,**c**) A375 wt and A375 HO-1 KO cells were treated with given ART concentrations for 24 h (**b**) and 48 h (**c**), respectively. Cell viability was measured via SRB assay. Respective DMSO (0.1%)-treated control was set at 100%. Data represent means ± SEM of three independent experiments (*n* = 3). The level of significance was calculated with Student’s *t*-test with with ns ≥ 0.05, * *p* < 0.05 and ** *p* < 0.01. (**d**) Graphical summary of the implication of HO-1 in ART-mediated cytotoxicity in melanoma cells.

The pharmacological modulation of HO-1 activity by ZnPP and Hmn demonstrated a clear implication of the enzyme activity on the toxicity of ART in melanoma cells. To prove this concept and to exclude any off-target effects apart from HO-1 by the used substances, HO-1 knock out (KO) cell clones were generated. A375 melanoma cells were transfected with plasmids, encoding endonuclease Cas9 and guide-RNAs specific for *HMOX1* (see Section 2). The complete loss of the HO-1 protein was confirmed via Western blot analysis. The most prominent clone was used for the experiments (Figure 6a). A375 wildtype (wt) cells and A375 HO-1 KO cells were treated in the same 24-well dish with the same concentrations of ART to ensure a high degree of comparability. The cell viability was measured via SRB assay and compared to the respective mock-treated (DMSO) control. After 24 h of treatment, ART was less toxic in A375 HO-1 KO cells than in A375 wt cells, even though no significance was detected via statistical analysis (Figure 6b). A similar effect was observed after 48 h of treatment. Again, ART was less toxic in A375 HO-1 KO cells than in A375 wt cells (Figure 6c). For this time point, statistical analysis resulted in a significant difference between melanoma cells with functional HO-1 and melanoma cells deficient in HO-1. Taken together, these data validate the ZnPP and Hmn data (Figure 5) indicating that the ART-mediated upregulation of HO-1 triggers the production of a toxic ART radical (Figure 6d).

## 4. Discussion

The introduction of immune checkpoint blockade strategies and molecularly targeted therapy in the last decade led to a substantial improvement in the survival rates of patients with malignant melanoma [48]. However, the battle against melanoma is far from won. Primary or acquired resistance against immunotherapy as well as targeted therapy is frequently observed [49,50,51]. Therefore, compounds with a selective mechanism towards cancer cells are in high demand. The “old drug” artesunate (ART) is an antimalarial agent recommended as first-line therapy against severe malaria by the WHO [52]. Moreover, several studies indicate that ART also displays a great antitumor capacity [8,9,10]. As the pharmacokinetic and toxicological characteristics of the drug are already well known, the approval process for cancer therapy could be less time- and cost-intensive, and melanoma patients could benefit from the repurposed drug. Before designing clinical trials and testing the anti-cancer properties of ART in human melanoma patients, further in vitro studies are needed to clarify the selectivity of the drug towards melanoma cells and its mode of action. In this study, we especially focused on the induction of oxidative stress and the implication of HO- 1 in the ART toxicity.

To analyze the selectivity of the drug, its effect on cell viability of malignant A375 melanoma cells and normal human dermal fibroblasts (NHDF) was compared. Treatment with ART (2.6–52 µM) resulted in a time- and dose-dependent decrease in the viability of the melanoma cells, whereas the viability of the NHDF was not affected by the same concentrations of the drug. This selective effect is also represented by the calculated IC_50_-values herein. Geng and colleagues detected noticeable higher IC_50_-values for ART over 48 h in melanoma cells: 56.54 µM in the OCM-1 cell line and 59.77 μM in the C918 cell line [11]. However, they analyzed uveal melanoma cells with distinct biological and clinical characteristics. In another study, the tumoricidal effect of ART was studied on 55 cell lines of the Developmental Therapeutics Program of the National Cancer Institute, USA. IC_50_-values ranging from 1.11 to 25.62 µM were detected, closely matching the results of our study [53]. One limitation of this study is the use of a single melanoma cell line (A375). In the future, additional studies with further melanoma cell lines should be conducted. The cytotoxic effect of ART on non-malignant cells is often ignored but a few studies analyzing this effect detected IC_50_-values in ovarian fibroblasts [54] and normal renal epithelial cells [55] which are similar to the IC_50_-values in NHDF from our study. One of the most decisive factors in drug development is the therapeutic index (also called “therapeutic ratio”) of a drug which describes the ratio between a plasma concentration causing a therapeutic effect and the concentration causing a lethal (animal studies) or toxic (human studies) effect [56]. In an analogous approach, we determined the in vitro therapeutic index by determining the ratio between the IC_50_-values for 96 h in A375 cells (therapeutic effect) and in NHDF cells (toxic effect), which was calculated to be approximately 19. Compared to the small therapeutic ratio of classical anti-cancer drugs such as Busulfan displaying an in vivo ratio of <2 [57], ART appears to have a remarkable therapeutic index.

Research on malaria and cancer therapy emphasizes that ART is activated in a Fe^2+^ catalyzed reaction [39,40]. The dependency on the divalent metal could explain the detected selectivity of the drug towards melanoma cells, as it is known that at least some cancer types display increased intracellular iron concentrations [26]. In our study, a significant “rescue” effect after co-treatment with ART and the iron-chelating agent deferoxamine (DFA) was observed, thus confirming the essential role of iron for the activation of the drug in melanoma cells. These data are in line with previously published studies which showed that iron is essential for the toxicity of ART in breast cancer cells [8], cervical cancer cells [42] and hepatocellular carcinoma cells [58]. Overall, the link between intracellular iron concentration and ART cytotoxicity is very interesting as it could be a basis for the development of new therapeutic approaches.

In addition to the increased intracellular iron concentrations, cancer cells display distinctly higher basal levels of ROS compared to their normal counterparts [17]. This makes them especially susceptible to an exogenous induction of ROS [18]. In order to elucidate the effect of ART on the modulation of ROS levels, A375 and NHDF cells were analyzed using DCFH-DA. In both cell lines, an increase in ROS was found after ART treatment. However, the extent of the increase was noticeably higher in melanoma cells. This is consistent with data from the literature, where ART increased oxidative stress in numerous cancer cell lines [24,42,43]. Data regarding the effects of ART on the redox state of normal (healthy) cells are less uniform and indicate cell type specificity [59,60].

An increase in oxidative stress initiated by ART results in unspecific oxidation of intracellular molecules [16]. Besides oxidative damage of essential biomolecules which can explain the cytotoxicity of the drug, redox-sensitive signaling proteins are also oxidized upon an induction of oxidative stress [61]. These “redox sensors” evolved to activate parts of the cellular antioxidative system [62]. One of the key redox-sensitive signaling proteins is Keap1 (kelch-like ECH-associated protein 1). The 69 kDa adapter protein is part of the Keap1-Nrf2 pathway, a major regulator of cytoprotective responses to endogenous and exogenous induction of ROS [63]. Upon oxidative stress, cysteine residues of Keap1 are oxidized causing its release from its binding partner Nrf2. Consequently, the transcription factor Nrf2 migrates to the nucleus, where it binds to antioxidative response elements (ARE) on the DNA, finally resulting in the transcription of several genes involved in the antioxidative response [63]. These genes encode proteins such as glutathione peroxidase 2 (GPx2), peroxiredoxin 1 (Pdrx1) and heme oxygenase 1 (HO-1) [64]. Therefore, the detected induction of HO-1 upon ART treatment in our study is not unexpected, as we measured a substantial increase in the ROS level caused by the drug. Previous studies also found that ART induces the expression of HO-1 by degrading Keap1 in head and neck cancer cells (HNC) [54] as well as in mice [65]. Nothing was previously known concerning melanoma cells.

Although there is some controversy, HO-1 is generally reported to be an antioxidative enzyme [66]. This is due to its enzymatic activity: the degradation of heme into the products biliverdin and CO [34]. Bilirubin, the reduction product of biliverdin, is considered to act as an antioxidant. Because of its assumed cytoprotective role, there are ongoing studies to inhibit HO-1 in cancer therapy [67]. In the context of the ART-mediated mechanism in cancer cells, it was shown earlier that high activity of Nrf2 (and thus high expression of HO-1) correlated with resistance against ART, and sensitivity to ART was increased upon the inhibition of Nrf2 [54,68]. These results are in line with the assumed protective role of HO-1. However, our data for melanoma cells indicate the exact opposite which may depend on another degradation product of heme iron. In the presence of ART, the pharmacological induction of HO-1 increased sensitivity towards the drug, while the pharmacological inhibition of HO-1 decreased sensitivity. In KO melanoma cells deficient in HO-1, ART was also less toxic than in wildtype melanoma cells. As described above, HO-1 catalyzes the degradation of heme into equimolar amounts of biliverdin, CO and Fe^2+^. The metal ion Fe^2+^ is known to be cytotoxic because of its prooxidative properties [66]. The induction of HO-1 was shown to increase the amounts of intracellular iron level [66]. Furthermore, Kwon and colleagues demonstrated that enhanced HO-1 activity accelerates the induction of Fe^2+^-dependent ferroptosis [32]. The Fe^2+^-releasing effect of HO-1 is especially interesting in the presence of the Fe^2+^-activated drug ART. Many studies, including our own, showed that the toxicity of ART is directly dependent on the intracellular iron level [8,42]. We would like to argue that ART induces a ROS-mediated HO-1 upregulation resulting in increased intracellular Fe^2+^ levels, which in turn triggers the generation of the toxic ART radical as part of an ongoing cycle (see Figure 6d). This result is in contrast with the above-mentioned studies from Roh and colleagues, and Hill and colleagues [54,68]. However, they investigated head and neck, and non-small-cell lung cancer cells, respectively, and mainly focused on the modulation of Keap1 and Nrf2. Nevertheless, further studies are still needed to finally clarify the involvement of HO-1 in the ART-initiated cytotoxicity in cancer cells.

## 5. Conclusions

In summary, ART selectively decreased the cell viability of melanoma cells without harming the studied fibroblasts. The toxicity of the drug seems to be highly dependent on the intracellular iron concentration, the induction of oxidative stress and upregulation of HO-1. Downregulation of ART-mediated HO-1 expression partly compensated the ART-mediated decrease in cell viability. This study emphasizes the potential of ART as a promising drug in the treatment of melanoma.

## Data Availability

The data presented in this study are available on request from the corresponding author.
